# Cytotoxic and apoptotic potential of some coumarin and 2-amino-3-carbonitrile selenophene derivatives in prostate cancer

**DOI:** 10.3906/kim-2008-56

**Published:** 2021-02-17

**Authors:** Metin YILDIRIM, Mehmet ERSATIR, Badel ARSLAN, Elife Sultan GİRAY

**Affiliations:** 1 Department of Pharmacy Services, Vocational School of Health Services, Tarsus University, Mersin Turkey; 2 Department of Chemistry, Arts and Science Faculty, Çukurova University, Adana Turkey; 3 Department of Stem Cell and Regenerative Medicine, Institute of Health Science, Mersin University, Mersin Turkey

**Keywords:** Coumarin, selenophene, antitumor agents, antioxidant activity, heterocycles

## Abstract

3-acetyl coumarin derivatives (1a-d) are formed as a result of condensation of salicylaldehyde derivatives and ethyl acetoacetate and were converted into coumarin-selenophene hybrid compounds (2a-d) in the basic medium by modified Gewald reaction in the presence of malononitrile and selenium. Products are characterized by nuclear magnetic resonance (NMR). The prepared compounds are screened for their anticancer activity against DU-145 cell line. In addition, selected target compounds are evaluated for apoptosis and oxidative stress on DU-145 (prostate carcinoma) cell lines.

## 1. Introduction

Cancer is a disease that occurs in any part of the body and spreads rapidly. It ranks second among the diseases that cause human deaths in the world. About 9 million people die each year from cancer. The most common types of cancer are breast cancer and colorectal cancer in women and lung cancer and prostate cancer in men. Although many drugs have been developed by scientists to treat cancer, they cause the cells to show resistance besides their fatal side effects. For this reason, demand for new cancer drugs acting with different mechanisms is increasing day by day.

Coumarin compounds are biologically active compounds that can be isolated from plants and also synthetically synthesized in laboratories [1]. Various studies have shown that Coumarin exhibit antiviral [2,3], antioxidant [4], anticonvulsant [5,6], anticancer [7–10], and also shows AcHE and BucHE enzyme inhibition activity [11–15]. Additionally, studies have shown that coumarin compounds showing cholinesterase enzyme inhibition are substituents in 3rd and 4th carbon and 6th and 7th carbons for this activity. Coumarins containing methoxy or hydroxy groups at the 6th and 7th carbons and containing acetyl, phenyl or methyl groups at the 3rd and 4th carbons are known to inhibit the acetylcholinesterase enzyme [16].

Selenophene compounds are five-membered heterocyclic aromatic compounds containing selenium element, which is very important and unique for the body. Proteins containing selenium are called 21st century protein (selenocysteine). It is known in the literature that compounds containing selenophene show biological activity such as anticancer [17–19], anti-HIV [20] and antifungal [21,22]. In addition, due to the high cytotoxic effect of compounds containing selenium, the new hybrid compounds that form with these compounds can be very effective and selective against cancer cells.

There are few studies on the synthesis of new compounds formed by combining coumarin and selenophene and investigating their biological activities. In 2017, Domracheva et al. synthesized the compounds of selenophenocoumarin and selenophenoquinolinone, examined in vitro cancer activities, and clarified the mechanism [23]. In 2020, Erşatır et al. synthesized 8 new coumarin-selenophene hybrid compounds and investigated the anticancer activities of these compounds and coumarins with starting compounds in the MCF-7 breast cancer cell line. They determined that the new compounds were more active than the starting materials [6]. By combining two or more pharmacofores with a single covalent bond and molecular architecture, we can minimize the side effects that may occur while increasing the efficiency and activity [24]. Most cancer medications such as Voreloxin, Cefatrizine, and Quarfloxin have also been created for this purpose. In the light of all this, the present study is to combine two effective and biologically active compounds with a single covalent bond to obtain compounds that will be effective against various cancers, particularly prostate cancer. The main purpose of this study is to examine the activities of coumarin compounds in different cancer lines and to test whether they show higher biological activity with the synergy created by combining hybrid compounds compared to the starting compounds. This study is a continuation of our previous study where we investigated the synthesis of eight coumarin-selenophene hybrid compounds and their antiproliferative activities on the MCF7 breast cancer cell line. Four of these compounds showed very good activity on this cell line. We examined the antiproliferative activities of these four compounds and their starting substances on the DU145 prostate cancer cell line. Also, Caspase 3, 8, and 9 activities of these four hybrid compounds and starting substances were measured, MDA (3,4-Methylene​dioxy​amphetamine), and glutathione levels were tested.

## 2. Materials and methods

### 2.1. Synthesis of coumarin and coumarin-selenophene derivaties

All coumarin and coumarin-selenophene derivatives were synthesized based on our previous study (Table 1) [4]. Their characterization studies were carried out by nuclear magnetic resonance (NMR) spectra, which is specified in supporting information.

**Table 1:** Synthesis of coumarin-selenophene hybrid compounds from coumarins.

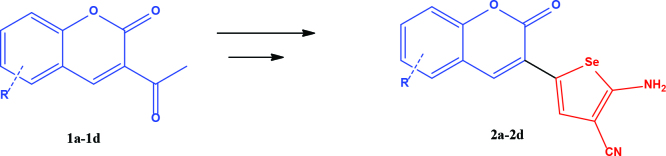		
Entry	Starting compounds	Product
1	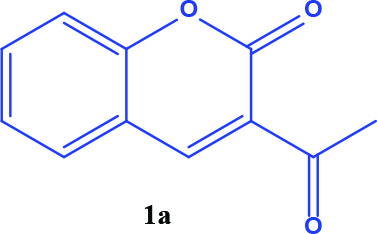	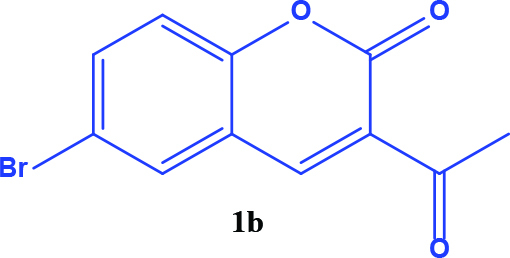
2	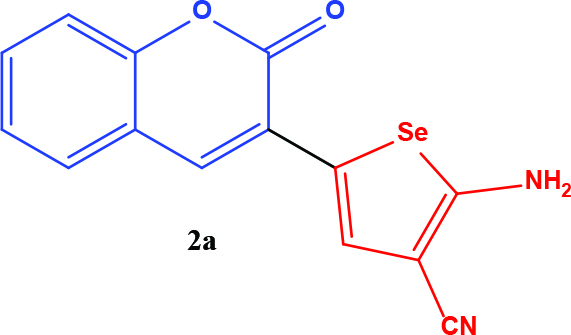	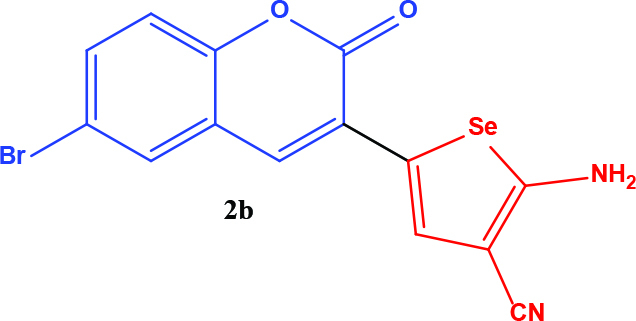
3	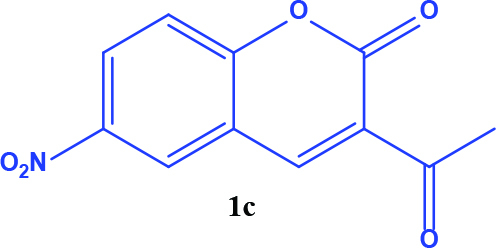	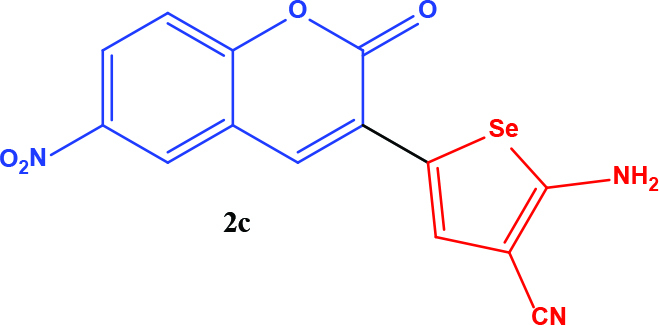
4	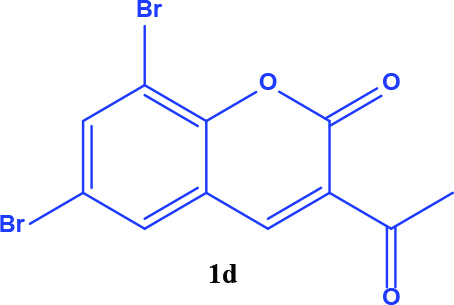	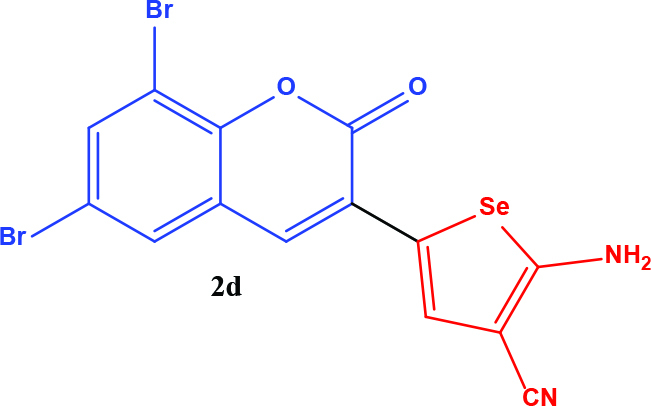

### 2.2. Antiproliferative activity

DU-145 human prostate cancer cell line was obtained from the American Type Culture Collection (ATCC, USA). Cells were routinely cultivated in RPMI 1640 supplemented with 10% fetal bovine serum, penicillin (100 U/cm^3^), and streptomycin (100 mg/cm^3^) at 37 °C and 5% CO_2_. Protein levels were measured by the Lowry method [25]. Cell line was selected based on many clinical trials showing the activity of selenium compounds in the reduction of prostate cancer [26].

The cells were seeded at a density of 3 × 10^4^cells per plate in 16 well E-Plate (xCeLLigence, ACEA Biosciences), then completed with medium and incubated overnight in a 5% CO_2_incubator at 37 °C. Subsequently, cells were treated with different tested (2a-2d, 1a-1d) at different concentration (0, 5, 10, 20, 40 µM). All experiments were performed in triplicate. Untreated cells were used as control groups. Doxorubicin (Sigma) was used as a reference drug.

### 2.3. Caspase 3, 8 and 9 assays

Caspase 3, 8, and 9 levels in DU-145 cell lysates were measured using Caspase 3- colorimetric assay kit (Cloud-Clone Corp., USA), Caspase-8 colorimetric assay kit (Cloud-Clone Corp., USA), and Caspase-9 colorimetric assay kit (Cloud-Clone Corp., USA). Assays were carried out according to the manufacturer’s instructions and then color changes were determined spectrophotometrically at 450 nm.

### 2.4. Lipid peroxidation measurement

MDA level of cell lysates were determined according to Ohkawa et al. The principle of the method depends on the measurement of the pink color produced by the interaction of thiobarbituric acid with MDA as a result of lipid peroxidation. The color density was measured via spectrophotmether at 532 nm. The results were expressed as nmol/mg protein [27].

### 2.5. Glutathione measurement

GSH levels were measured using the method reported by Beutler et al. The reaction of glutathione and DTNB (Ellman’s reagent), generated 2-nitro-5-mercapto-benzoic acid. The color density was measured via spectrophotometer at 412 nm. The results were showed as μmol/mg protein [28].

## 3. Results and discussion

Coumarin compounds belong to benzopyrone family of plant secondary metabolites and very important for their therapeutic potential in cancer treatments. Their derivatives have shown wide variety of biological activities such as antioxidant, antiproliferative, antimicrobial, antiinflammatory, and antituberculosis. Although chemotherapy has been shown to be the most appropriate option against to cancer, it has been known to have deleterious systemic side effects. In order to avoid these side effects, new drug candidate needs to be designed [29,30]. In this study, novel potential anticancer agents were synthesized and tested on prostate cancer which is second most common cancer among men worldwide. Then, caspase enzyme activities and antioxidant properties were determined.

**Antiproliferative activity**

The significance of coumarin-selenophene derivatives shows antiproliferative activity against prostate cancer [31]. The cytotoxicity of coumarin and coumarin-selenophene derivatives was determined by xCELLigence system. Cell proliferation was determined by differences in cell-impedance variations when treated tested compounds on DU-145 cells. The results of xCELLigence assay IC_50_values are summarized in Table 2 and Table 3.

**Table 2:** Antiproliferative effects of coumarin compounds (1a-1d) on MCF7 breast cancer and DU145 prostate cancer cell line.

	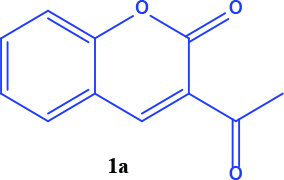	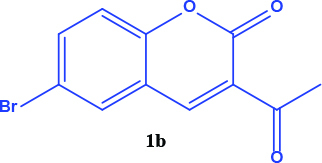	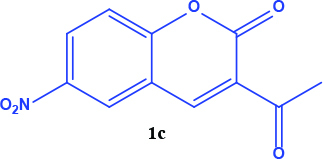	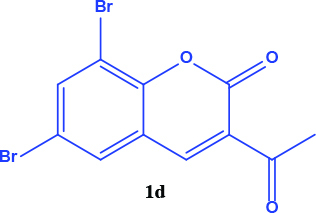
MCF7*	85.45 µM	43.91 µM	65.74 µM	25.20 µM
DU145	48.00 µM	53.00 µM	55.00 µM	59.00 µM

*MCF7 results were given in the previous study.

**Table 3:** Antiproliferative effects of coumarin-selenophene compounds (2a-2d) on MCF7 breast cancer and DU145 prostate cancer cell line.

	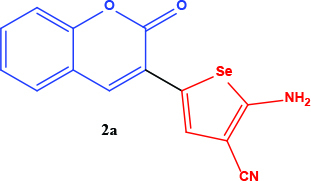	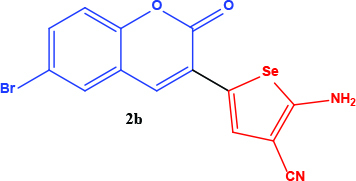	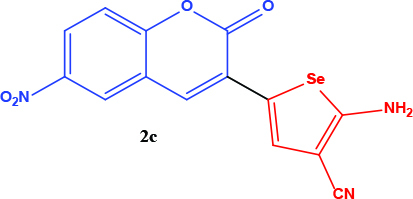	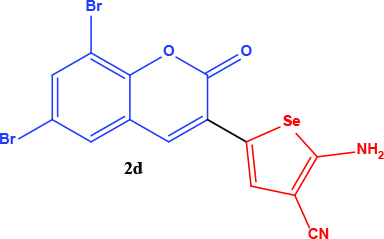
MCF7*	11.73 µM	10.83 µM	12.21 µM	15.18 µM
DU145	20.00 µM	36.00 µM	39.00 µM	44.00 µM

*MCF7 results were made in the previous study.

Coumarin compounds (1a-1d) have higher IC_50_values ​​compared to the hybrid compounds (2a-2d). (IC_50_= 48.0 - 59.0 µM for coumarins and 20.0 - 44.0 µM for hybrid compounds). According to this result, the biological activity of the hybrid compounds was better than the starting compounds that formed it.

The hybrid compound 3-acetyl coumarin-selenophene without substituents on the phenyl ring (2a) appears to be the most active compound in the DU145 cell line. (IC_50_= 20.0 µM) When the effect of the substituents was examined; the IC_50_value was 36.0 µM when Br was on the 6th carbon and 39.0 µM when nitro was present. It was observed that the IC_50_value of the hybrid compound containing bromine at the 6 and 8 carbons is the lowest (IC_50_= 44.0 µM).

It appears that hybrid compounds that are active even from the reference substance used in the previous study are also active in the DU145 cell line. Hybrid compounds also appear to be active in the DU145 cell line from the self-forming starting compounds. While the hybrid compound without substituents in the phenyl ring is the most active compound, the activity decreases in the presence of electron withdrawing groups at the 6th carbon. Myers et al. reported that 5 days of coumarin treatment inhibits the proliferation of two malignant prostate cell lines (DU145 and LNCaP) [32].

7-hydroxycoumarinyl gallate ester showed high antiproliferative activity, which was superior to gallic acid, against DU-145 cell line [33].

In conclusion, the cytotoxic activity of the coumarin–selenophenes (2a-2d) and coumarin derivatives (1a-1d) against DU-145 cancer cell line: 2a>2c>2b>2d>1a>1c>1b>1d. When coumarin–selenophenes (2a-2d) were compared with their related coumarin derivatives (1a–1d), among them, 2a (IC_50_= 20 ± 1.2 mM) is the most potent candidate to suppress proliferation of prostate cancer cells.

**Apoptosis**

**Figure 1:**
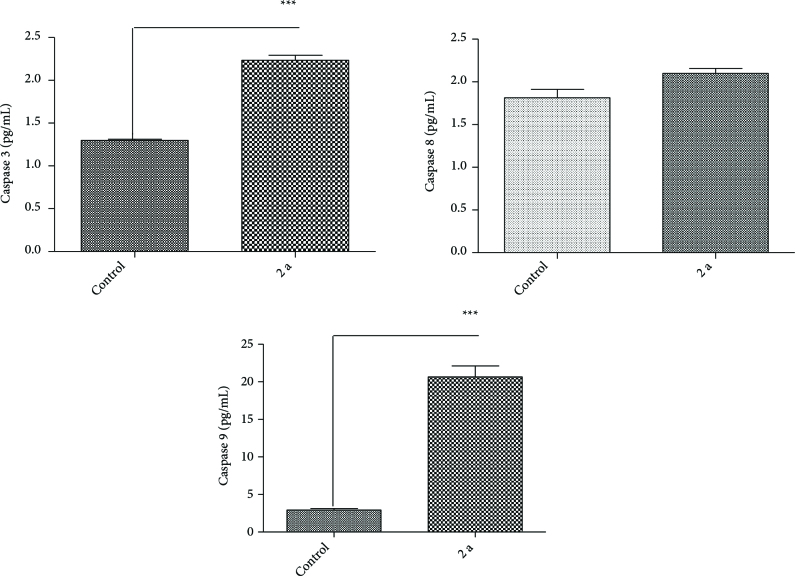
Effect of 2a on Caspase 3, 8, and 9 level in DU-145 cancer cells treated with IC_50_ concentration. *** P < 0.05, compare with control

To disclose the molecular mechanism that is involved with apoptosis inducement, we measured Caspase 3, 8, and 9 levels in DU-145 cells treated with various concentration of coumarin (1a-1d) and coumarin selenophene derivatives (2a-2d) by ELISA method. The expression of Caspase 8 is involved in extrinsic pathway of apoptosis. Furthermore, the activation of Caspase 9 is a characteristic feature of intrinsic apoptosis pathways. Caspase 3 and 9 expression levels increase after treatment with 20.0 mM of 2a was statistically significant (Figure 1). Caspase 8 level also increased but it was not significant. Other tested compounds did not exhibit significant effect on Caspase 3, 8, and 9. According to the data were obtained 2a induced on the extrinsic pathway of apoptosis (Figure 1). Umar et al. have reported that 4-flourophenylacetamide-acetyl coumarin induced apoptotic cell death by ROS-evoked p53-mediated caspase-dependent pathway in A549 cells [34].

**Biochemical analysis**

Ersatir et al. showed that coumarin-selenophene derivatives have antiproliferative activity against to MCF-7 breast cancer line [4]. Increased oxidative stress is related to weakening of the cellular defence mechanism. Cancer cells generate reactive oxygen species (ROS) more than healthy cells. Several drugs used in cancer therapy kill cancer cells by ROS production, and today many researchers are working on developing natural or synthesized cancer therapeutic agents that increase ROS production. High concentration of reactive radicals causes lipid peroxidation, protein modification, and DNA damage causing apoptosis or necrosis.

**Figure 2:**
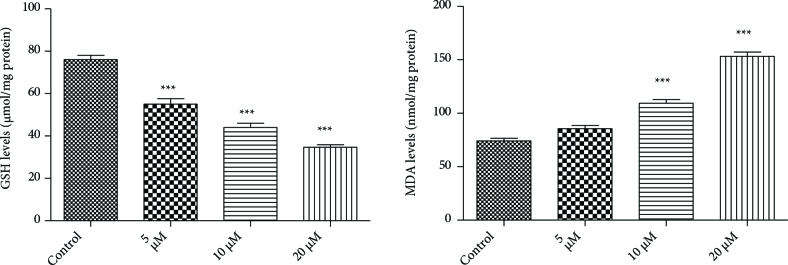
Effect of 2a on GSH and MDA levels in DU-145 cell line. *** P < 0.05, compare with control.

Malondialdehyde level is the most popular and reliable markers of oxidative stress and the antioxidant status in cancerous patients. In this study,2a significantly increased MDA levels in a dose-dependent manner (Figure 2). Hacioglu et al. proved that ZnSO_4_caused a concentration-dependent increase in oxidative stress, apoptosis [35].

Kar et al. demonstrated that there is a correlation between increased oxidative stress and decreased cell growth and induction of apoptosis [36].

Kim DH et al. reported that ROS generation leads to apoptosis in human colon cancer HCT116 cells [37]. GSH has a tripeptide structure, which is an important antioxidant in cytosol.

GSH has many cellular functions such as antioxidant defence via direct interaction with ROS or via activities of detoxication enzymes like GSH peroxidases and GSH-S-transferases.

GSH depletion trigger to initiating an oxidative stress. Intracellular GSH levels are very important for cell death mechanism. In this study, we showed correlation between descending concentration of 2a compound and GSH levels (Figure 2). Interestingly, GSH can regulate Caspase 3 and 9 catalytic activity as well as their proteolytic activation [38].

Supplementary Materials
