# Supplementary material for: Cytotoxic and apoptotic potential of some coumarin and 2-amino-3-carbonitrile selenophene derivatives in prostate cancer
Source: Turk J Chem. 2021 Feb 17;45(1):192–8. doi: 10.3906/kim-2008-56 (PMC7955924; doi:10.3906/kim-2008-56)

Supporting Information

Contents:

**I**	Instrumentation Details	**S2**
**II**	^{1}H and ^{13}C NMR spectra of compounds **1a-1d**	**S3-S6**
**III**	^{1}H and ^{13}C NMR spectra of compounds **2a-2d**	**S7-S10**

I. Instrumentation Details

Unless noted otherwise, all of compounds were used as provided without further purification. All of compounds were obtained from Merck and Sigma-Aldrich.

^{1}H and ^{13}C NMR spectra were recorded in $CDCI_3$ or $DMSO_4$-d_6 [using the solvent peak as internal reference ($DMSO_4$-d_6 : δ H 2.50; δ C 39.51 and $CDCl_3$ at 7.27 ppm for ^{1}H and 77.0 ppm for ^{13}C) on a Bruker 300 MHz Ultrashield TM spectrometer operating at 300 MHz and 75 MHz, respectively or a Bruker Avance III 400 MHz spectrometer operating at 400 MHz and 100 MHz, respectively. All chemical shift values are quoted in ppm and coupling constants quoted in Hz. Multiplicities are indicated, s (singlet), d (doublet), t (triplet), q (quartet), sept (septet), m (multiplet), br s (broad singlet). Follow up of the reactions and checking the purity of the compounds were made by TLC on silica gel-precoated aluminium sheets (Type 60, F_{254}, Merck, Darmstadt, Germany) using hexane/ethyl acetate 80–20 (4:1, v/v) and the spots were detected by exposure to UV lamp at λ254 nanometer for few seconds.
The chemical names given for the prepared compounds are according to the IUPAC system.

IR spectra were recorded on a Perkin-Elmer 55148 spectrometer.
Melting points were determined using an Electrothermal 9100 instrument.
Elemental analyses were measured on a Thermo Flash 2000 Organic Elemental Analyzer.

I. ^{1}H and ^{13}C NMR spectra of compounds (1a-1d)

^{13}C NMR spectra of compound **1a**

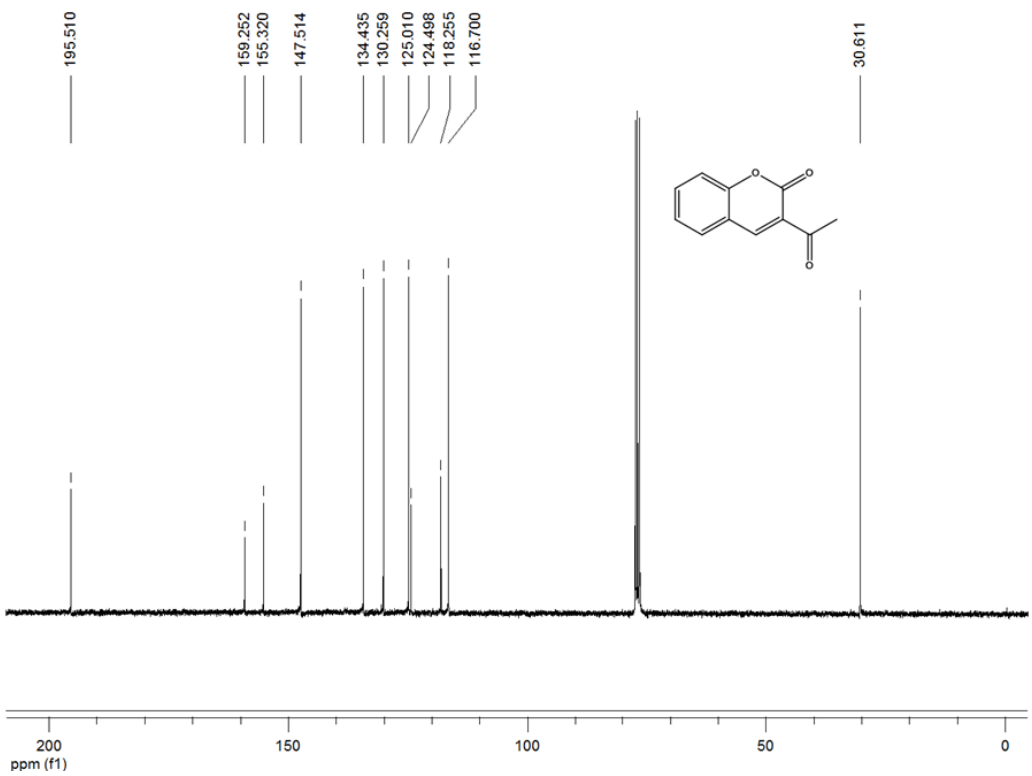

^{1}H NMR spectra of compound **1a**

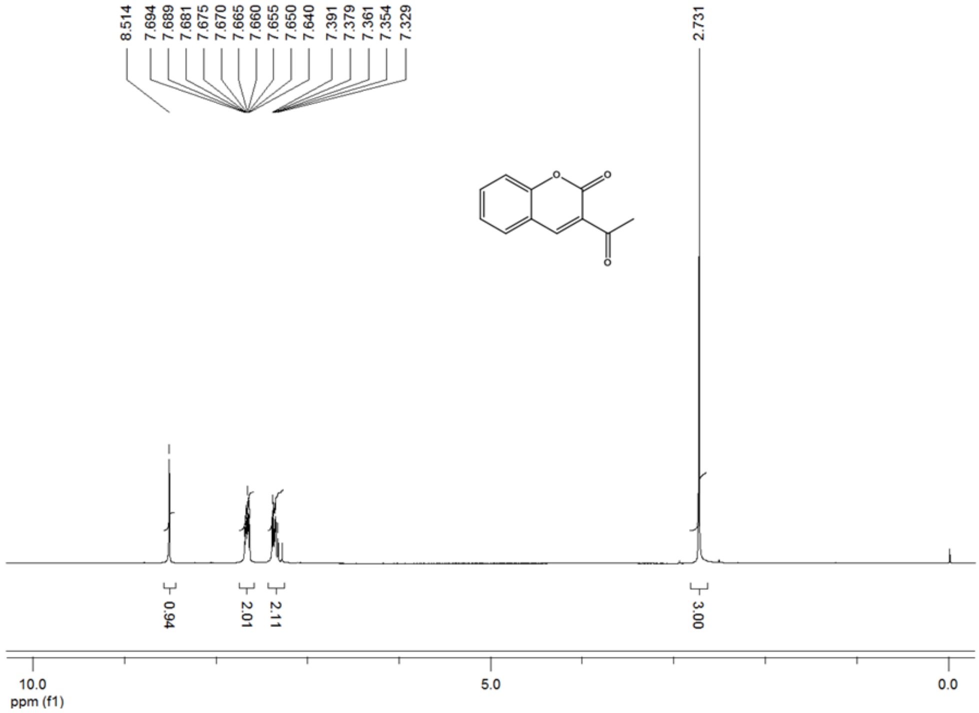

^{13}C NMR spectra of compound **1b**

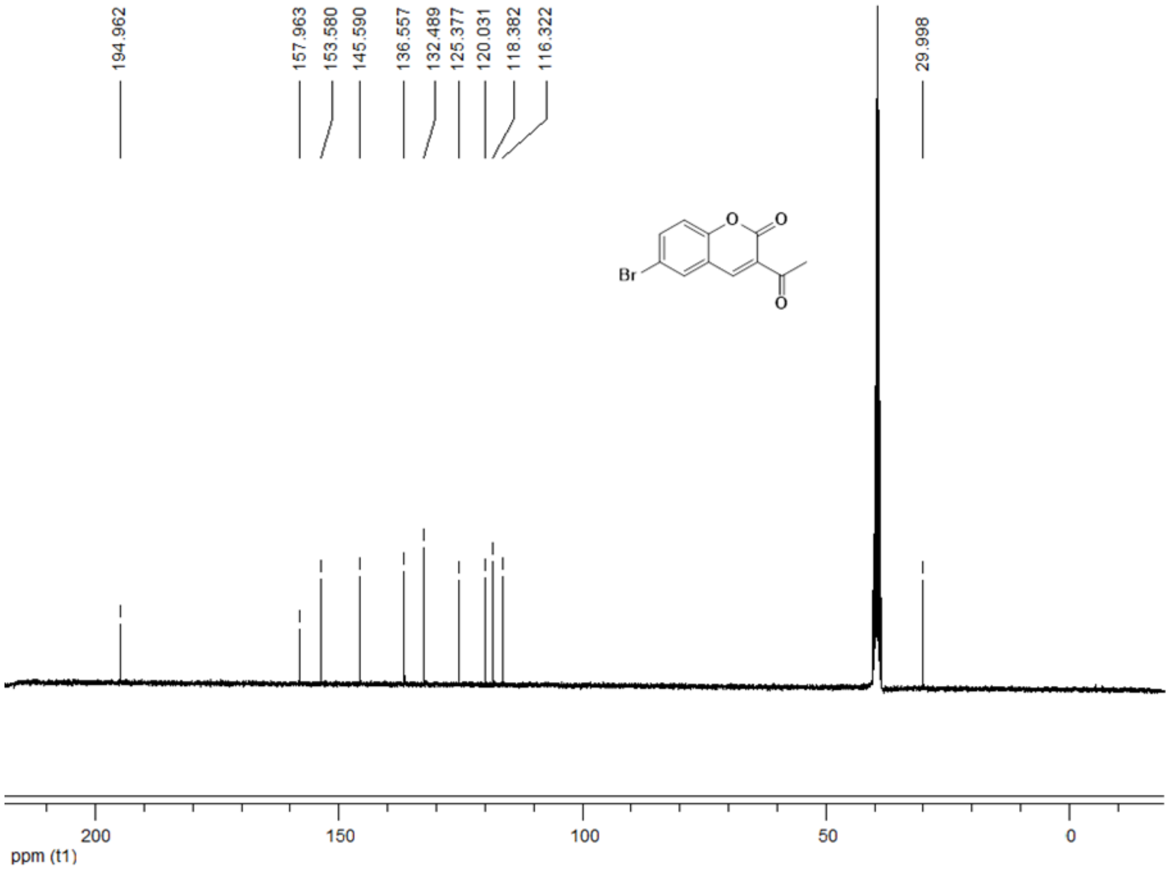

^{1}H NMR spectra of compound **1b**

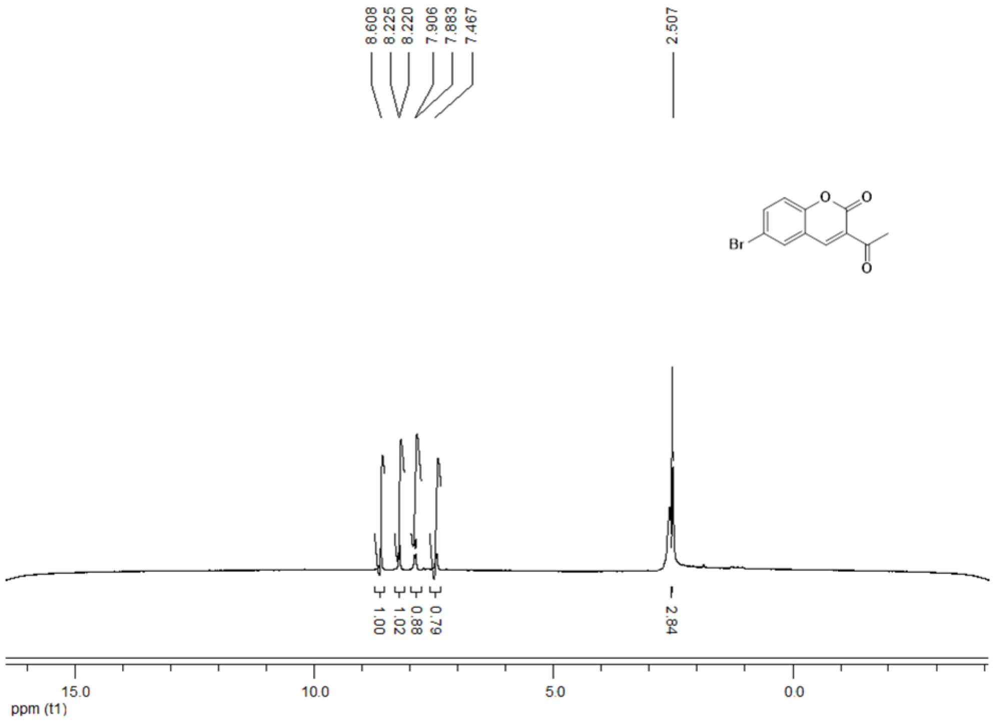

^{13}C NMR spectra of compound **1c**

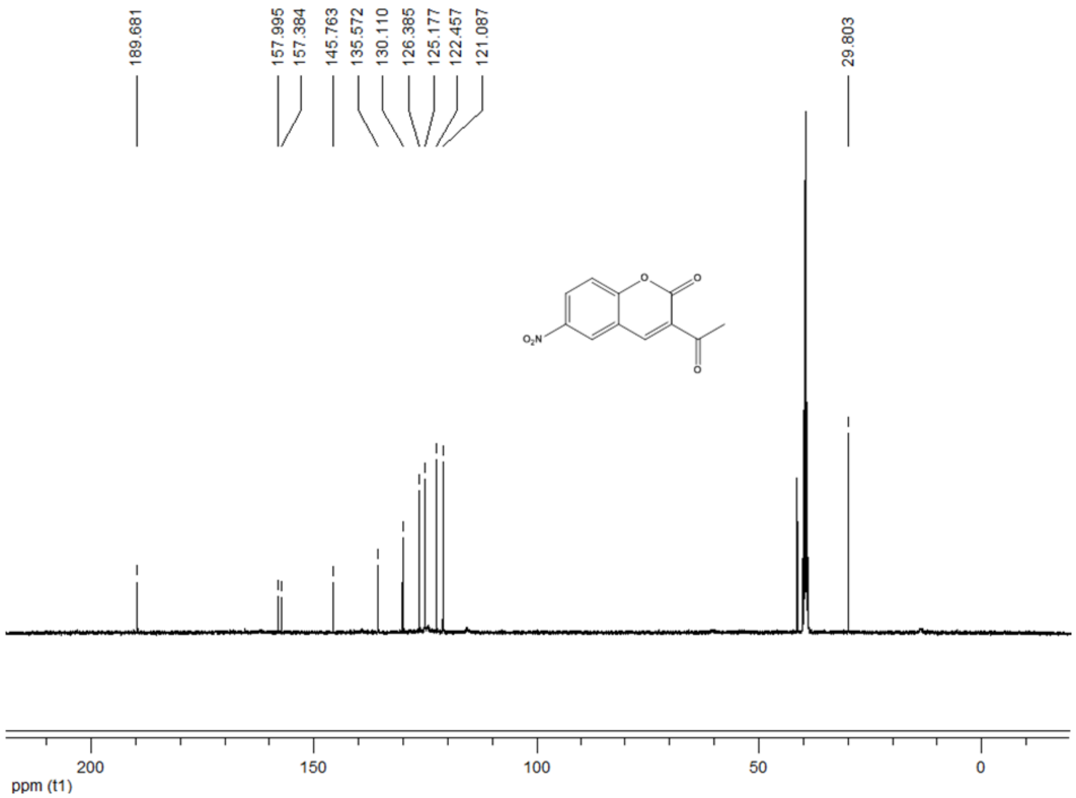

^{1}H NMR spectra of compound **1c**

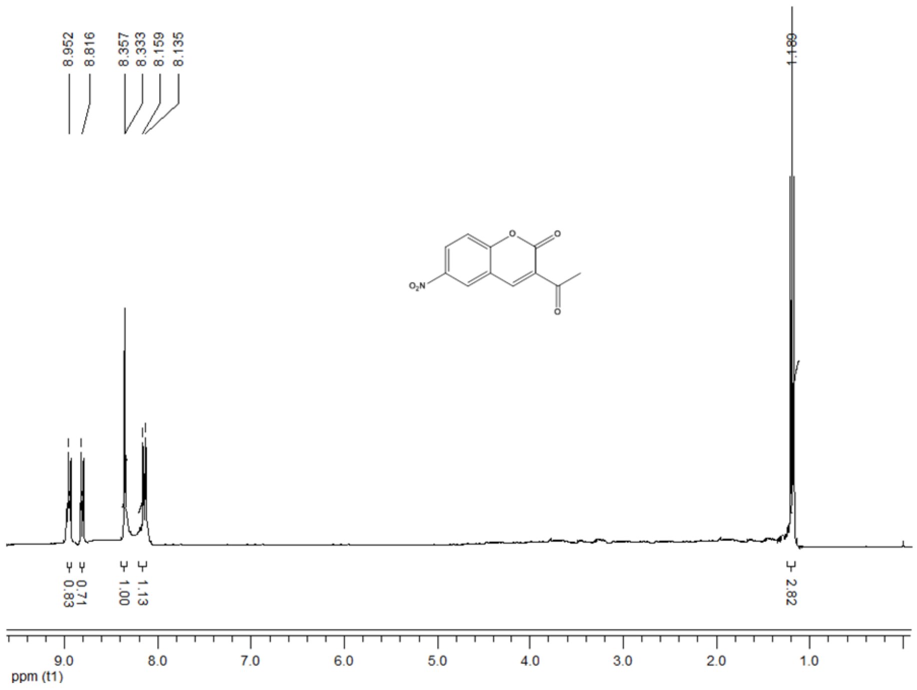

^{13}C NMR spectra of compound **1d**

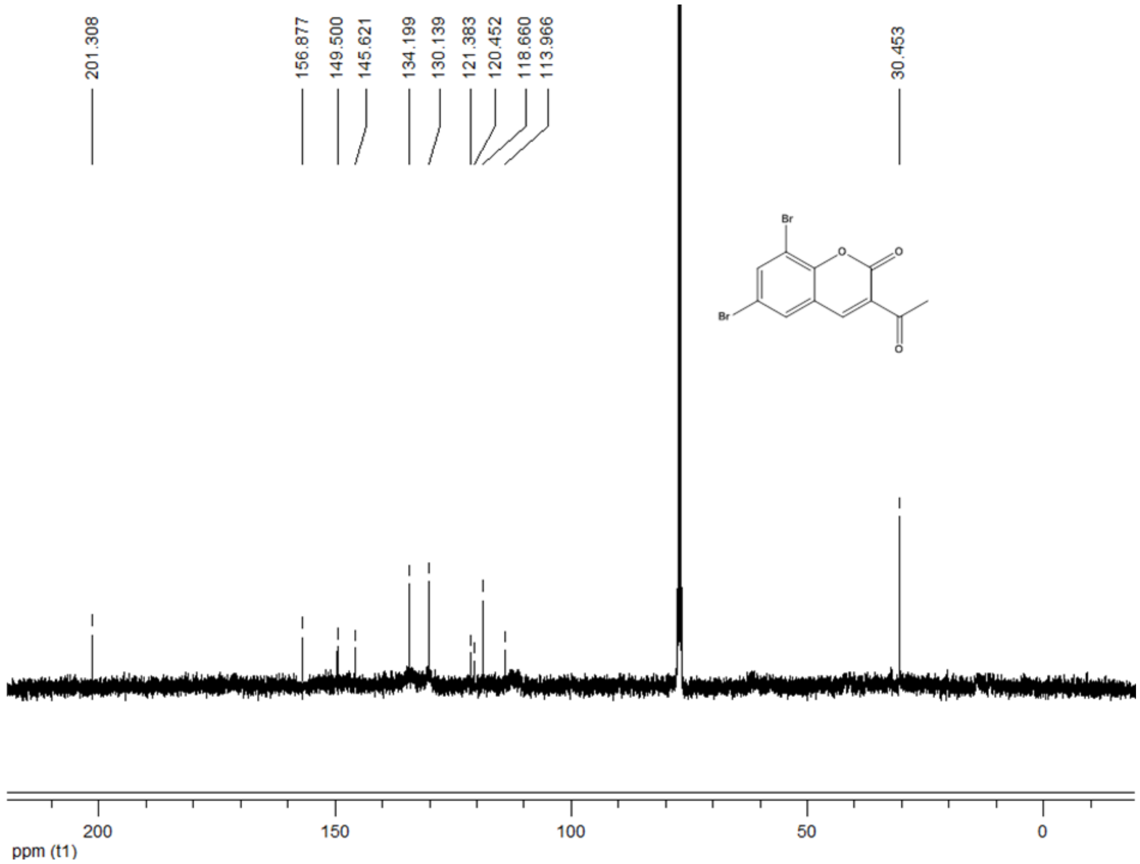

^{1}H NMR spectra of compound **1d**

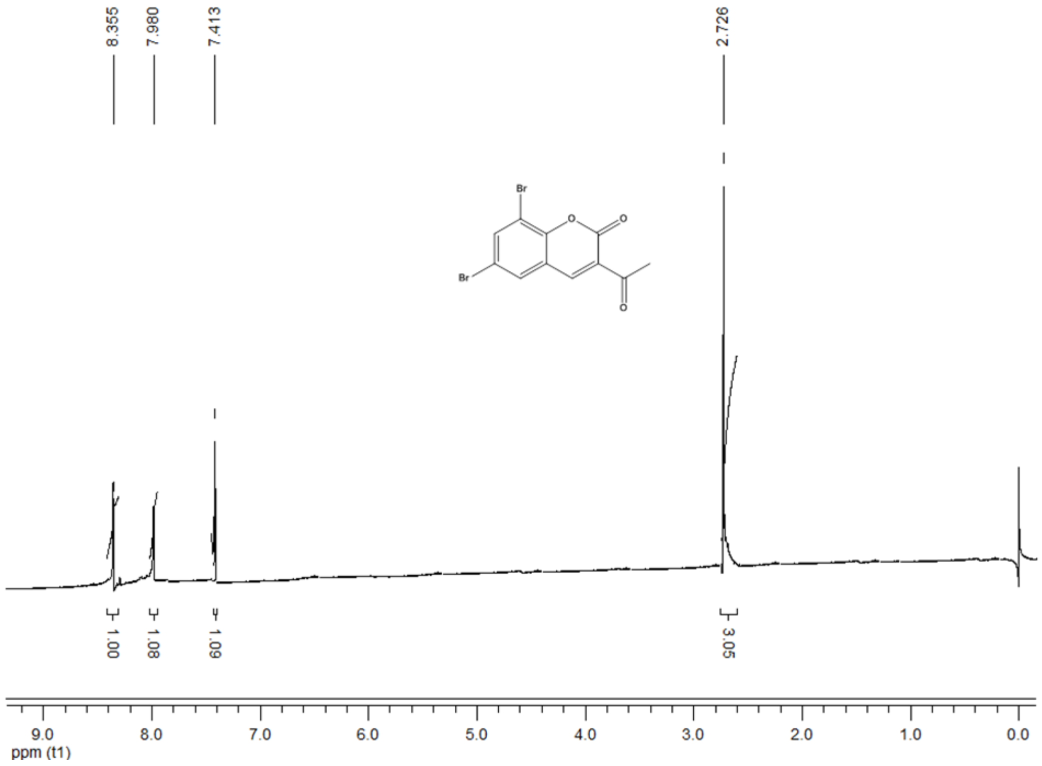

II. ^{1}H and ^{13}C NMR spectra of compounds (2a-2d)

^{13}C NMR spectra of compound **2a**

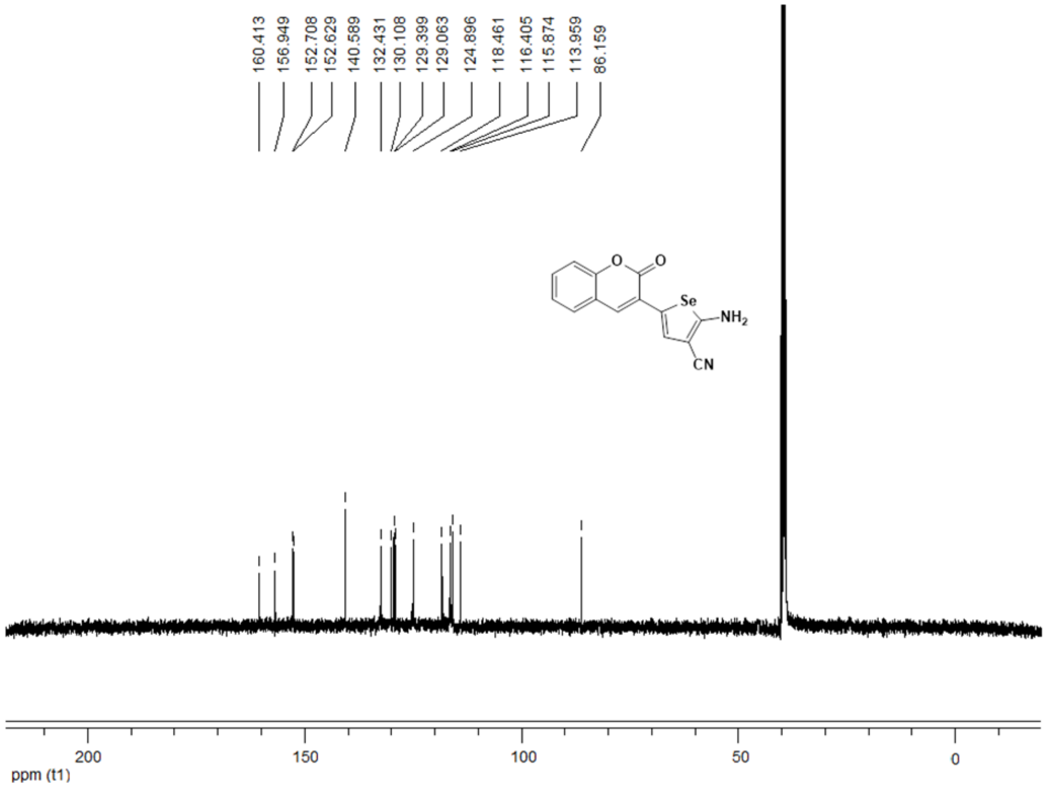

^{1}H NMR spectra of compound **2a**

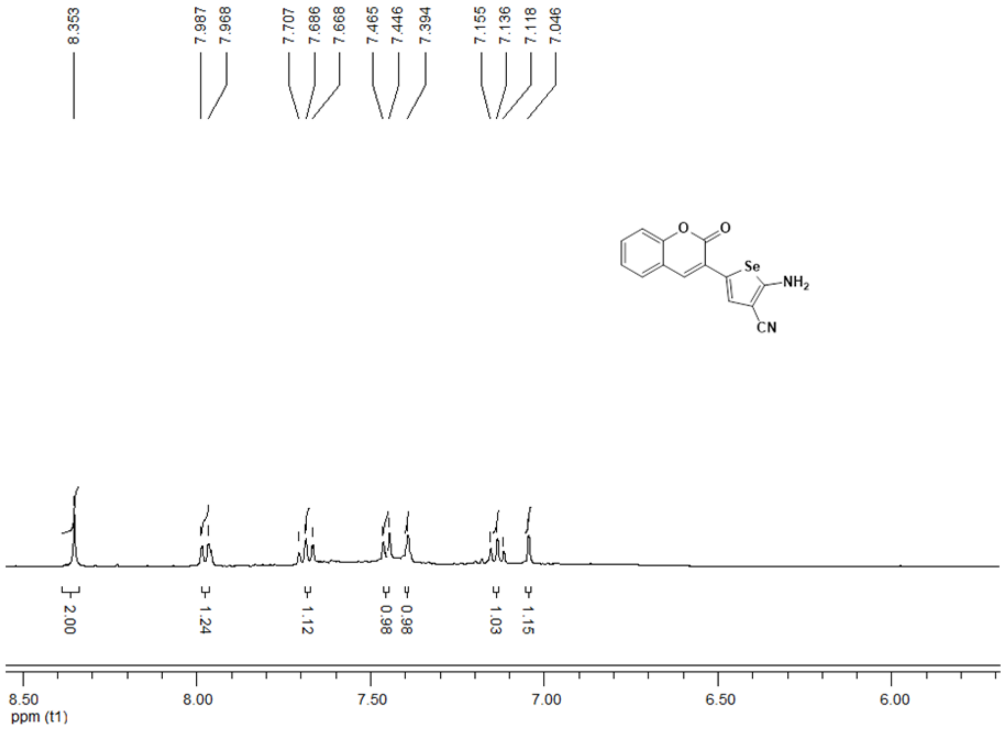

^{13}C NMR spectra of compound **2b**

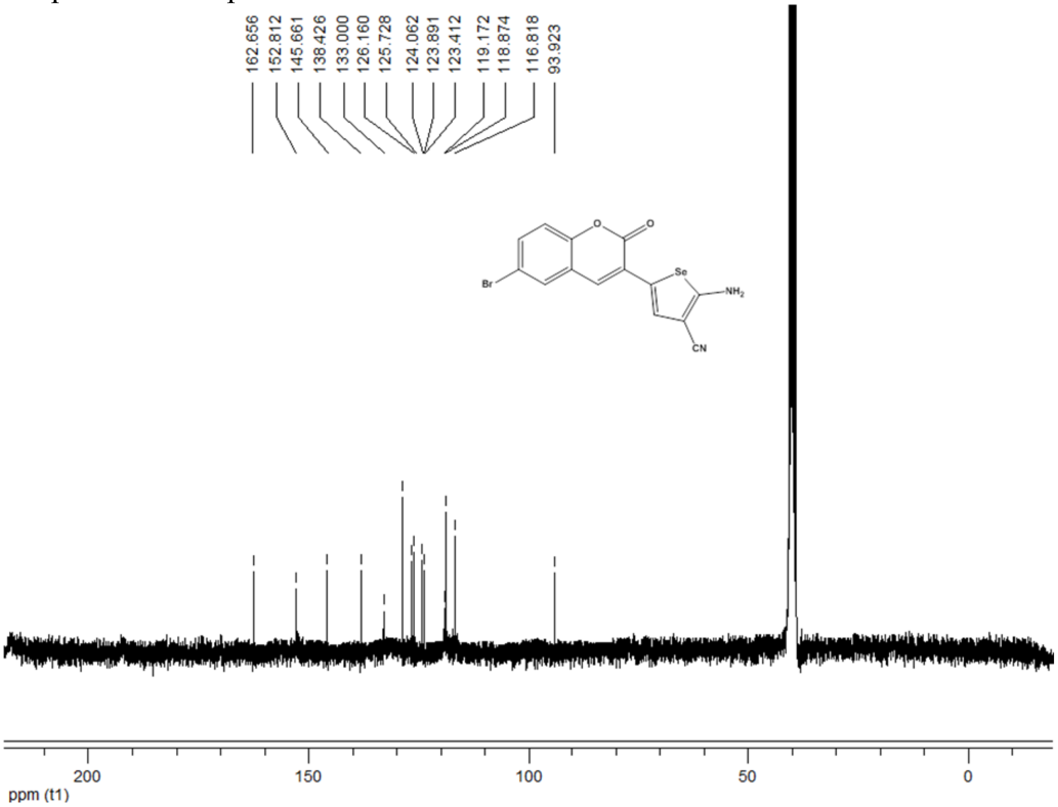

^{1}H NMR spectra of compound **2b**

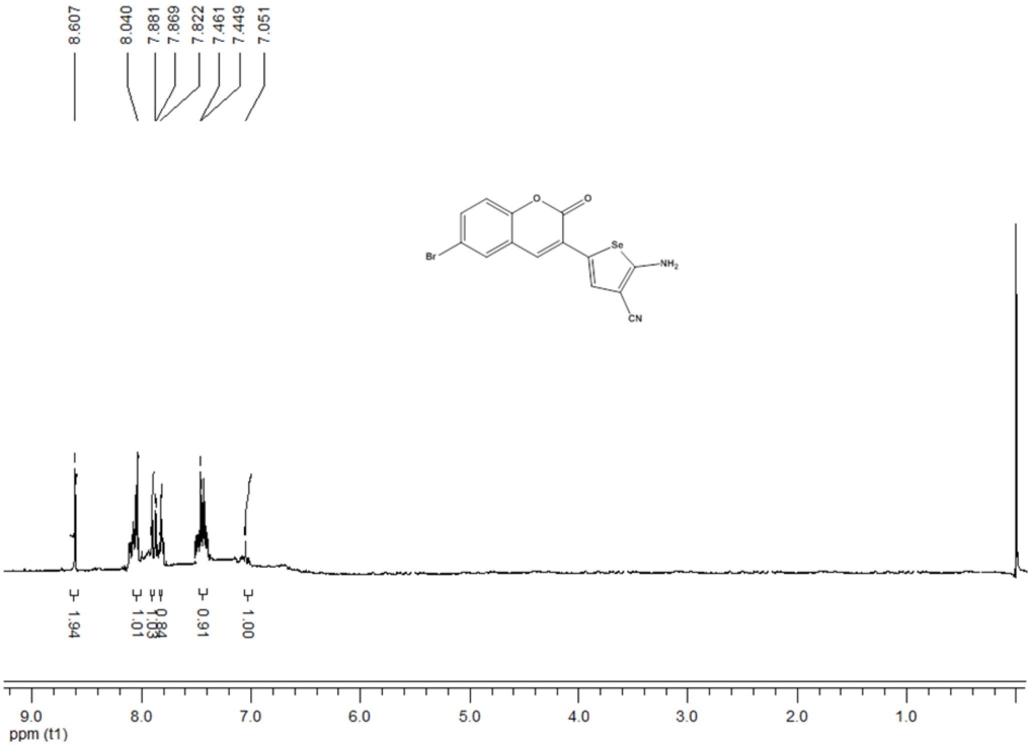

^{13}C NMR spectra of compound **2c**

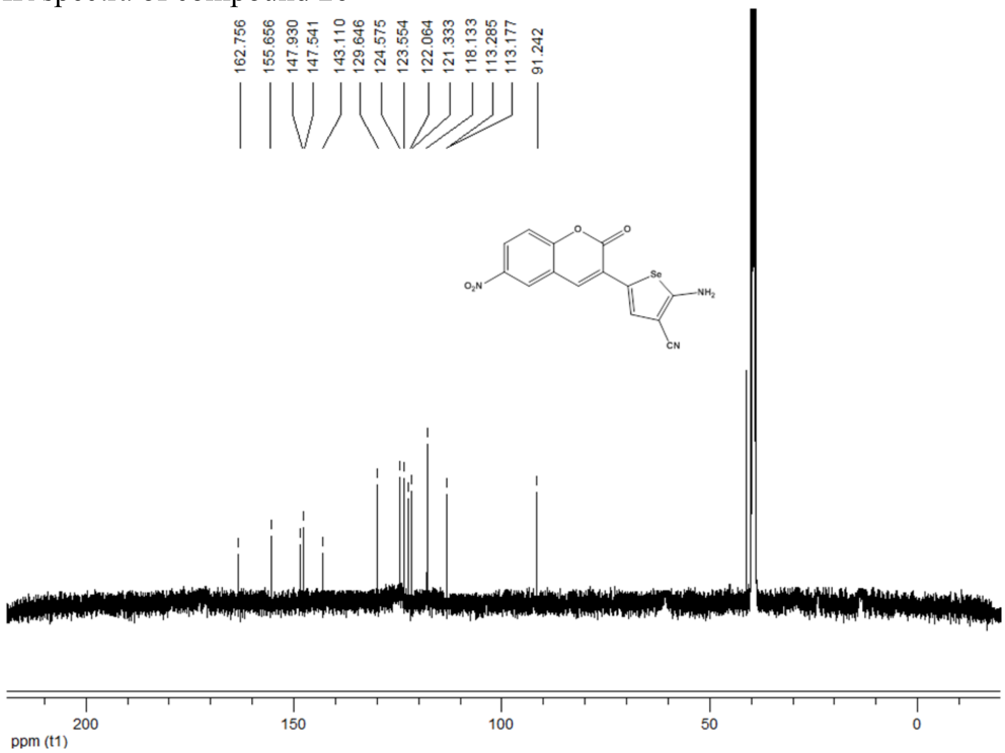

^{1}H NMR spectra of compound **2c**

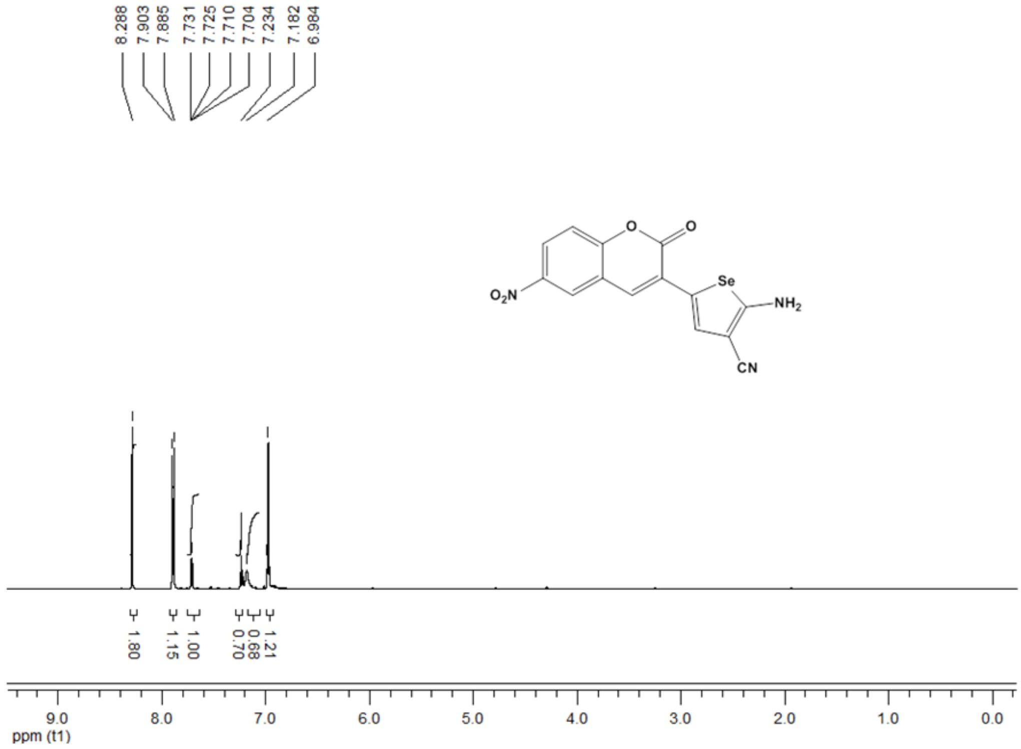

^{13}C NMR spectra of compound **2d**

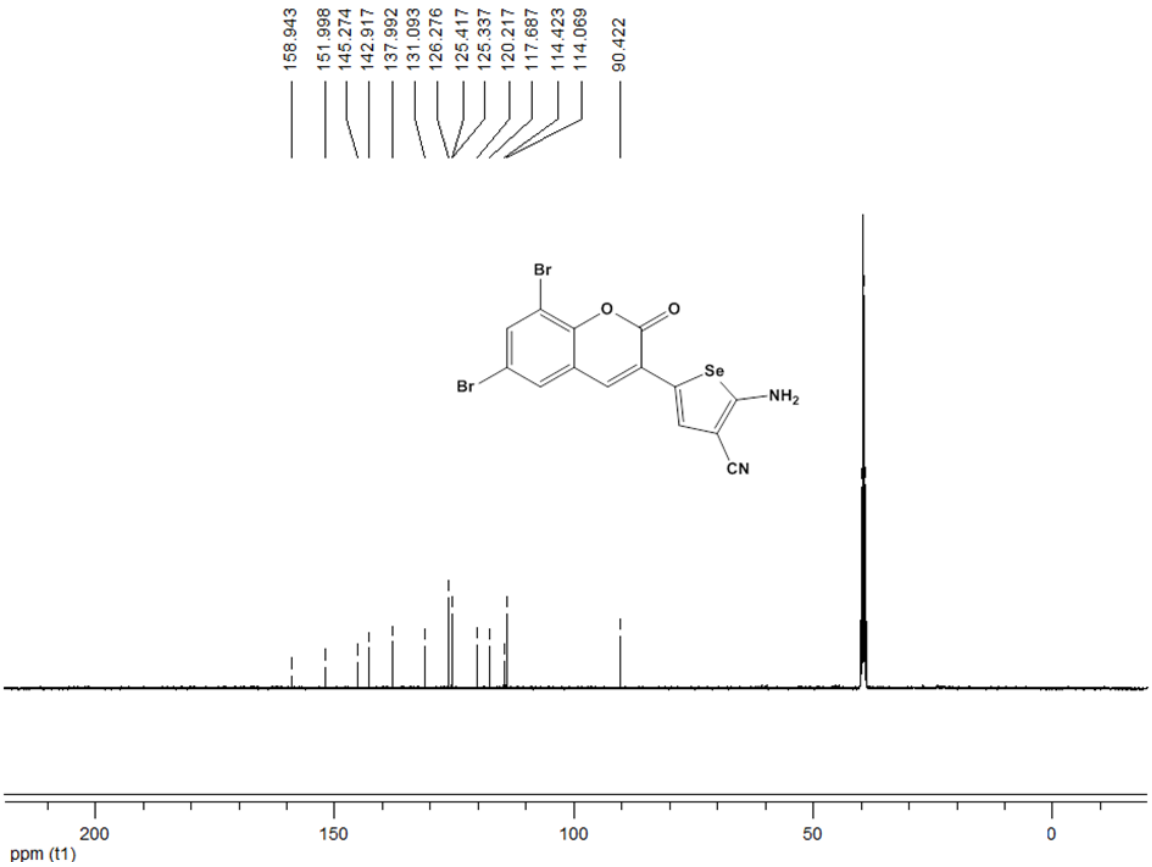

^{1}H NMR spectra of compound **2d**

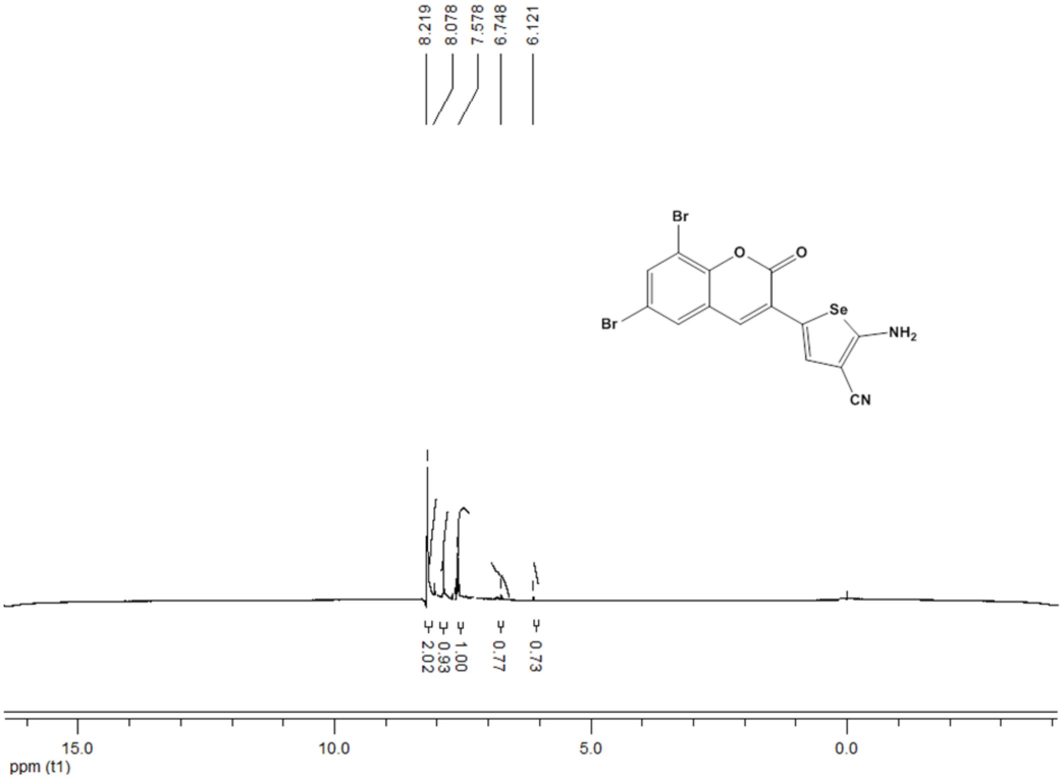

Supplement: Supplementary file 1 — Supplementary Materials [file turkjchem-45-192-sup001.pdf]
